# The Effects and Mechanism of Quercetin Dietary Supplementation in Streptozotocin-Induced Hyperglycemic Arbor Acre Broilers

**DOI:** 10.1155/2020/9585047

**Published:** 2020-02-10

**Authors:** Linlin Ying, Maria Tabassum Chaudhry, Fenglin Xiao, Yanjun Mao, Mi Wang, Bo Wang, Shanshan Wang, Yao Li

**Affiliations:** ^1^Institute of Animal Nutrition, Northeast Agricultural University, Harbin 150030, China; ^2^Faculty of Veterinary Sciences, Bahauddin Zakariya University, Multan 60800, Pakistan

## Abstract

Quercetin, a flavonoid found in fruits and vegetables, is widely distributed as a secondary metabolite in the plant kingdom. Oxidative stress plays a role in the pathogenesis of diabetes mellitus (DM). The present study investigated the effects of quercetin dietary supplementation on streptozotocin- (STZ-) induced hyperglycemic Arbor Acre (AA) broilers by determining the levels of fasting blood glucose (FBG), fasting insulin (FINS), biochemical indicators, oxidative stress markers, inflammatory cytokines content, antioxidant enzymes activities in tissues, and mRNA expression of genes relating to the insulin signaling pathway. Three hundred one-day-old healthy AA broilers were randomly assigned into 5 treatments; A, control healthy broilers; B, STZ-induced broilers; C, STZ-induced broiler dietary supplemented with 0.02% quercetin; D, STZ-induced broiler dietary supplemented with 0.04% quercetin; and E, STZ-induced broiler dietary supplemented with 0.06% quercetin. The results showed that quercetin supplementation relieved the side effects of STZ-induced oxidative stress by changing activities of antioxidant enzymes, decreasing malondialdehyde (MDA) and nitric oxide (NO) levels, activating expression of genes relating to PI3K/PKB signaling pathway that modulate glucose metabolism and reduce oxidative damage, thereby decreasing FBG and increasing FINS levels. These findings suggest that quercetin exhibits a protective effect in STZ-induced hyperglycemic AA broilers via decreasing oxidative stress.

## 1. Introduction

Diabetes mellitus (DM) is a disease characterized by hyperglycemia, resulting in severe metabolic imbalances and nonphysiologic changes in many tissues [1]. One of the major hypotheses proposed to explain the hyperglycemia-induced onset of diabetic complications is an increase in the rates of oxidative stress [2]. Many chronic inflammations are very likely involved in the pathogenesis of insulin resistance and type II diabetes [3]. Oxidative stress (OS) is involved in the pathogenesis of DM partly via reactive oxygen species (ROS) generation in diabetic human and animal models [4]. Several researchers have examined oxidative stress markers in diabetic rats and found increased ROS levels in pancreatic islets [5]. The balance between ROS and antioxidants is a major mechanism in preventing the damages from oxidative stress. Increased ROS generation and inflammation lead to insulin resistance [6].

Various experimental diabetes models have been proved to induce animal characteristic function and structural changes. These models include rats and mice that become diabetic after the administration of alloxan and STZ [7, 8]. A limited study has been reported in the area of AA broiler diabetes. STZ is a DNA alkylating agent [8], and its intraperitoneal or intravenous injection caused marked infiltration of inflammatory cells and reduced insulin secretion leading to diabetes [9–11]. STZ may increase the production of ROS and inhibit enzyme activities related with the scavenging of free radical [12]. Intraperitoneal administration of STZ (45-55 mg/kg body weight) decreased insulin sensitivity and induced hyperglycemia and diabetes mellitus in mice and rats [13, 14]. At the same time, several studies have been conducted with natural antioxidants for the prevention of oxidative damage under high oxidative stress conditions. The major enzymatic antioxidants directly involved in neutralization of ROS and reactive nitrogen species (RNS) are comprised of superoxide dismutase (SOD), catalase (CAT), and glutathione peroxidase (GSH-Px) [15, 16]. The nonenzymatic antioxidants include endogenous and exogenous antioxidants [17–19]. Quercetin, a major flavonoid, is found abundantly in fruits, vegetables, legumes, and green tea [20]. It has physiological properties, i.e., antioxidation, anti-inflammation, antipathogenicity, antivirus, antimicrobial effect, anticancer, and cardioprotection [21–24].

The present study was conducted to confirm the relationship between oxidative stress, glucose metabolism, and insulin signaling pathway in STZ-induced AA broilers. In addition, we explored the effects of quercetin dietary supplementation on the levels of biomarkers related to oxidative stress in AA hyperglycemic broilers. At the same time, an effort was made to understand the underlying mechanisms of these actions.

## 2. Materials and Methods

### 2.1. Main Reagents

Quercetin (purity ≥ 97%) and STZ (purity ≥ 98%) were purchased commercially from Sigma-Aldrich Chemical Company, USA. The commercial kit for FBG, FINS, AST, ALT, NO, MDA, MCP-1, IL-6, TNF-*α*, SOD, GSH-Px, and CAT were purchased from Nanjing Jiancheng Bioengineering Institute, Nanjing, Jiangsu, P.R. China.

### 2.2. Test Animals

One-day-old Arbor Acres (AA) broiler chicks were purchased from Harbin Yinong Poultry Industry.

### 2.3. Experimental Induction of Hyperglycemia/STZ Diabetes in AA Broilers

Hyperglycemia in AA broilers was induced chemically using STZ via intraperitoneal route at a dose of 50 mg kg^−1^ body weight; the injection was provided once a week for two weeks at the beginning of the trial. The normal control group was injected with the same volume of isotonic NaCl.

### 2.4. Care and Use of Animals

All procedures used in this study were approved by the Animal Care and Use Committee of the Northeast Agricultural University. Housing, management, and care of the birds were in accordance with the guidelines of Agricultural Animal in Agricultural Research and Teaching of Heilongjiang Province (HEI Animal Management Certificate No. 11928).

### 2.5. Experimental Design

A total of 300 one-day-old Arbor Acres broilers with similar body weight (36.78 ± 1.41 g) were purchased from a local hatchery and randomly allocated into 5 groups with 6 replicates and 10 broilers in each replicate (60 broilers per treatment). The assigned groups were as follows: A, normal control broilers (NC); B, STZ-induced broilers (HC); C, STZ-induced broilers dietary supplemented with 0.02% quercetin (basal diet + 0.2 g quercetin/kg feed); D, STZ-induced broilers dietary supplemented with 0.04% quercetin (basal diet + 0.4 g quercetin/kg feed); and E, STZ-induced broilers dietary supplemented with 0.06% quercetin (basal diet + 0.6 g quercetin/kg feed). Quercetin dehydrate powder was mixed with basal diet and offered in a mash form (5 mm) after grinding (Table 1).

### 2.6. Experimental Diets

Corn-soybean meal based diets were formulated according to Chinese Broiler Feeding Standards (NY/T33-2004) to meet the nutrient requirements of AA broiler chickens (Table 2).

All chickens were raised under the same management and environmental conditions. 16 L : 8D lighting was provided during the experiment. Water and experimental diets were available ad libitum, followed by routine immunization. Chickens were inspected daily for any health-related problems. The experimental study lasted for 42 days.

At the end of experiment, 2 chickens per replicate were randomly selected and blood was collected from the jugular vein in a sterile test tube for serum biochemical analysis. Serum was separated from the blood by centrifuging at 3500 rpm at 4°C for 15 min and was stored at -20°C until biochemical analyses. Then the chickens were slaughtered; the liver and pancreas were excised out, washed in ice-cold normal saline, patted dry and frozen in cryopreserved tubes in liquid nitrogen, and stored at -80°C.

### 2.7. FBG, FINS, Biochemical Indicators, and Oxidative Stress Marker Assay

The levels of FBG were measured using a colorimetric method. The FINS levels were determined using a UV-Vis spectrophotometer (UV1206). Contents of aspartate aminotransferase (AST), alanine aminotransferase (ALT), and MDA were determined using colorimetry. The colorimetry was performed by an enzymatic reader using commercially available diagnostic kits (Nanjing Jiancheng Bioengineering Institute, Nanjing, Jiangsu, P.R. China) following the manufacturer's instructions. For serum and liver NO levels, 150 *μ*L of the serum and liver sample homogenate were mixed with 20 *μ*L of Griess Reagent and 130 *μ*L of deionized water in a microplate. The mixture was incubated for 30 min at room temperature. After the incubation, the sample absorbance was measured at 548 nm using *μ*Quant ELISA Reader (Tecan Instruments, Austria).

### 2.8. Inflammatory Cytokine Assay

The serum inflammatory cytokines including monocyte chemoattractant protein-1 (MCP-1), interleukin-6 (IL-6), and tumor necrosis factor-*α* (TNF-*α*) were determined using commercially available enzyme linked immunosorbent assay (ELISA) (Nanjing Jiancheng Bioengineering Institute, Nanjing, Jiangsu, P.R. China) following the user guide provided with kits. The optical density (OD) value of each sample was detected at the wavelength of 450 nm with a Bio-Rad microplate reader (Tecan Instruments, Austria).

### 2.9. Antioxidant Enzyme Assay

The liver and pancreatic tissues were used for assaying activities of CAT, SOD, and GSH-Px using colorimetric kits with an F-4500 Fluorescence spectrophotometer (Hitachi, Japan) according to the instructions of the commercial kits (Nanjing Jiancheng Bioengineering Institute, Nanjing, Jiangsu, P.R. China).

### 2.10. mRNA Expression of Genes

Total RNA was extracted using a TRIzol reagent (Invitrogen Corp., Carlsbad, CA, USA), and cDNA was synthesized using a PrimeScript 1st strand cDNA synthesis kit (Takara Bio Inc., Kyoto, Japan). RT-qPCR was performed as previously described [25]. According to a gene bank, the primers were designed by Oligo 6.0 software and Primer premier 5.0 software (Table 3).

### 2.11. Statistical Analysis

The experimental data were analyzed by a general linear model using SPSS 20.0 statistical package for Windows (2010, SPSS Inc., Chicago, IL 60606-6307). The difference and interaction among treatments were examined by a one-way ANOVA with Tukey's test. Calculated △Ct (corrected sample) = the mean value of the target gene − the mean value of the internal reference gene; △△Ct = △Ct − the mean value of the control group. Differences among treatment means with a probability level of *P* < 0.05 were accepted as statistically significant, and all the results were expressed as the “mean values ± standard deviation”.

## 3. Results

### 3.1. Effects of Quercetin on FBG and FINS Levels in STZ-Induced AA Broilers

**Figure 1:**
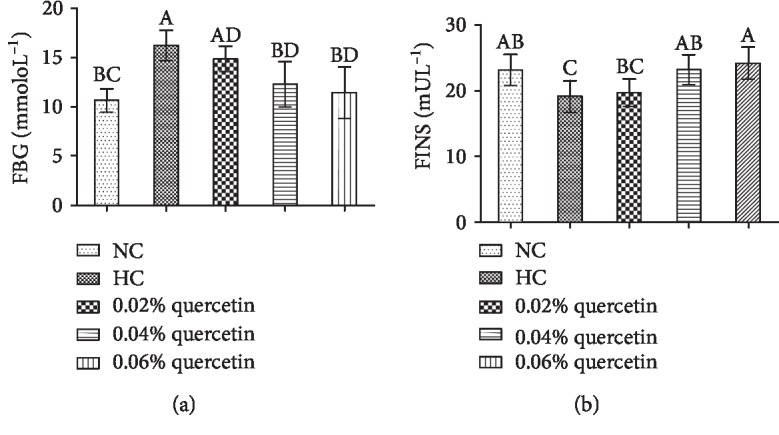
The effects of quercetin dietary supplementation on fasting blood glucose (FBG) and fasting insulin (FINS) levels in STZ-induced AA broilers. NC (normal control broilers), HC (STZ-induced broilers), and quercetin (0.02%, 0.04%, and 0.06%) groups. Statistical analysis was performed by one-way ANOVA followed by Tukey's test. Values are expressed as the means ± standard deviation (S.D.) and *n* = 10. Values with different letters are significantly different ((a) *P* < 0.01 and (b) *P* < 0.05).

The FBG and FINS levels of normal broilers were 10.63 ± 1.20 mmol · L^−1^ and 23.18 ± 2.37 mU · L^−1^, respectively. STZ significantly increased the FBG levels of broilers (34.4%, *P* < 0.01) that were decreased by 0.04% and 0.06% quercetin dietary supplementation (24.43% and 29.58%, respectively; *P* < 0.01) (Figure 1(a)). On the other hand, STZ administration significantly decreased FINS levels compared to that of normal broilers (17.4%, *P* < 0.05) and dietary supplementation with 0.04% and 0.06% quercetin significantly increased FINS by 17.58% and 20.85%, respectively (*P* < 0.05) (Figure 1(b)).

### 3.2. Effects of Quercetin on Content of Serum Biochemical Indicators in STZ-Induced AA Broilers

**Figure 2:**
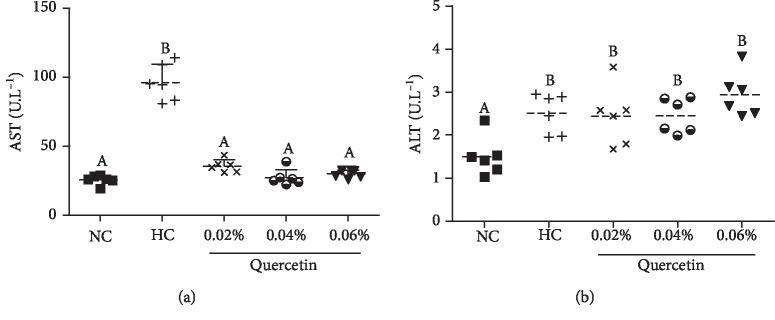
The effects of quercetin dietary supplementation on the content of serum aspartate aminotransferase (AST) and alanine aminotransferase (ALT) in STZ-induced AA broilers. NC (normal control broilers), HC (STZ-induced broilers), and quercetin (0.02%, 0.04%, and 0.06%) groups. Statistical analysis was performed by one-way ANOVA followed by Tukey's test. Values are expressed as the means ± S.D. and *n* = 10. Values with different letters are significantly different ((a) *P* < 0.01 and (b) *P* < 0.05).

The content of serum AST and ALT in normal broilers was 25.43 ± 3.37 U · L^−1^ and 1.50 ± 0.46 U · L^−1^, respectively. In STZ-induced broilers, the content of AST (73.54% increase, *P* < 0.01) and ALT (40.51% increase, *P* < 0.05) was significantly increased when compared with normal broilers. Dietary supplementation with 0.02%, 0.04%, and 0.06% quercetin significantly decreased serum AST content compared to STZ-induced diabetic broilers (by 63.02%, 68.92%, and 66.3%, respectively; *P* < 0.01); however, there was no significant decrease in serum ALT content of STZ-induced broilers (*P* > 0.05) (Figure 2).

### 3.3. Effects of Quercetin on the Levels of Oxidative Stress Markers in STZ-Induced AA Broilers

#### 3.3.1. Effects of Quercetin on the Levels of Serum NO and Liver NO in STZ-Induced AA Broilers

**Figure 3:**
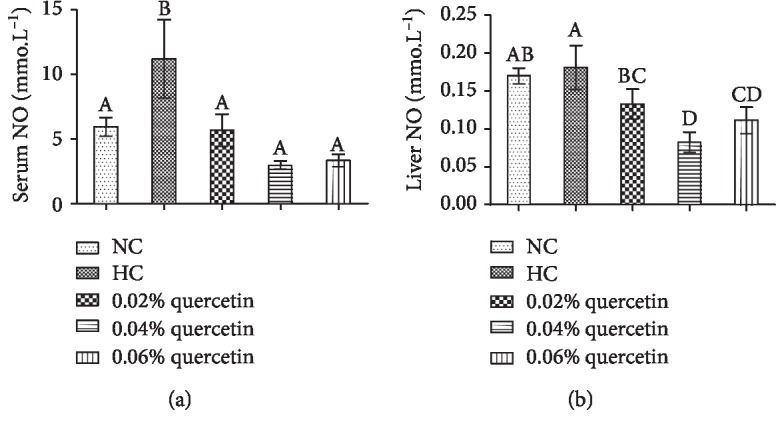
The effects of quercetin dietary supplementation on the levels of serum nitric oxide (NO) and liver NO in STZ-induced AA broilers. NC (normal control broilers), HC (STZ-induced broilers), and quercetin (0.02%, 0.04%, and 0.06%) groups. Statistical analysis was performed by one-way ANOVA followed by Tukey's test. Values are expressed as the means ± S.D. and *n* = 10. Values with different letters are significantly different (*P* < 0.01).

The levels of serum NO and liver NO in normal AA broilers were 5.95 ± 0.71 mU · L^−1^ and 0.17 ± 0.01 mU · L^−1^, respectively. In STZ-induced broilers, the serum NO levels were significantly increased by 46.9% (*P* < 0.01) and compared with the STZ-induced broilers, quercetin dietary supplementation at the three different levels significantly decreased serum NO levels by 49.2%, 73.3%, and 70.1%, respectively (*P* < 0.01) (Figure 3(a)). STZ administration did not significantly increase liver NO levels as compared to normal broilers (*P* > 0.05) and quercetin dietary supplementation at the levels of 0.02%, 0.04%, and 0.06% significantly decreased liver NO levels by 26.6%, 54.4%, and 38.4% (*P* < 0.01), respectively, when compared with STZ-induced broilers (Figure 3(b)).

#### 3.3.2. Effects of Quercetin on Serum MDA Content in STZ-Induced AA Broilers

**Figure 4:**
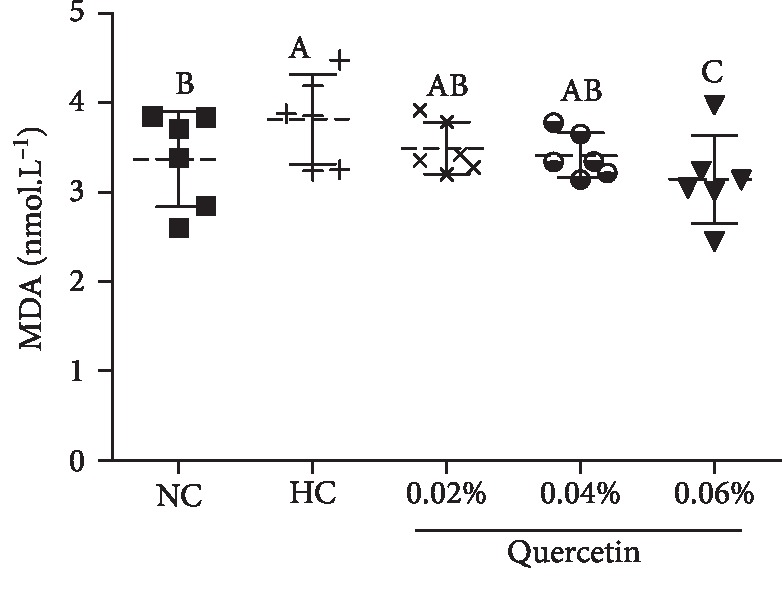
The effects of quercetin dietary supplementation on serum malondialdehyde (MDA) content in STZ-induced AA broilers (nmol L^−1^). NC (normal control broilers), HC (STZ-induced broilers), and quercetin (0.02%, 0.04%, and 0.06%) groups. Statistical analysis was performed by one-way ANOVA followed by Tukey's test. Values are expressed as the means ± S.D. and *n* = 10. Values with different letters are significantly different (*P* < 0.01).

In STZ-induced broilers, serum MDA was significantly increased as compared to normal broilers (11.8%, *P* < 0.01) and quercetin dietary supplementation at the level of 0.06% significantly decreased MDA levels by 17.8% (*P* < 0.01) (Figure 4).

### 3.4. Effects of Quercetin on Content of Inflammatory Cytokines in STZ-Induced AA Broilers

As compared to the normal broilers, the content of serum MCP-1 and IL-6 was not significantly higher in STZ-induced broilers (*P* > 0.05). On the other hand, quercetin dietary inclusion at the level of 0.04% significantly increased serum TNF-*α* content by 34.4% (*P* < 0.01), and these TNF-*α* levels were significantly higher than those of the controls (Table 4).

### 3.5. Effects of Quercetin on Activities of Antioxidant Enzymes in the Liver and Pancreas of STZ-Induced AA Broilers

Comparing with NC, STZ administration had no significant effect on the liver activity of SOD (*P* > 0.05). However, quercetin at the level of 0.04% significantly decreased activities of GSH-Px by 22.9% and increased CAT by 8.7% compared with STZ-induced broilers (*P* < 0.01) (Table 5).

Comparing with NC, STZ administration had significant effects on activities of pancreas SOD and GSH-Px (*P* < 0.05), whereas it had no significant effect on CAT (*P* > 0.05). Quercetin treatments effectively decreased SOD (*P* < 0.05) and CAT (*P* < 0.01) activities, while it had no significant effect on GSH-Px activities (*P* > 0.05) compared with STZ-induced broilers. 0.02% quercetin dietary supplementation significantly decreased CAT activities (36.97%, *P* < 0.01), and 0.06% quercetin dietary supplementation was the most effective in decreasing the activities of SOD (27.2%, *P* < 0.05) (Table 6).

### 3.6. Effects of Quercetin on mRNA Expression of Genes Relating to the PI3K/PKB Insulin Signaling Pathway

Compared with NC, expression of IR, PI3K, and PKB/Akt mRNA was significantly increased by 26.2%, 37.1%, and 29.9% in STZ-induced broilers (*P* < 0.001), and expression of GSK-3*β* and IRS-1 mRNA was changed (28% increase, *P* > 0.05; 26.7% decrease, *P* > 0.05, respectively). Dietary supplementation with 0.02% and 0.04% quercetin significantly downregulated mRNA expression of IR, PI3K, PKB/Akt, and GSK-3*β* (*P* < 0.01) and also significantly upregulated expression of IRS-1 mRNA (*P* < 0.05). Among the three levels of quercetin, 0.04% quercetin worked best in affecting mRNA expressions of IR, IRS-1, PI3K, PKB/Akt, and GSK-3*β* (Table 7). These results indicated that quercetin dietary supplementation regulated the PI3K/PKB signaling pathway in STZ-induced AA broilers.

## 4. Discussion

We found that quercetin dietary supplementation improved serum FBG, FINS, AST, and inflammatory cytokine content of TNF-*α*, as well as oxidative stress status in the liver and pancreas of STZ-induced hyperglycemic AA broilers. To our knowledge, the current study was the first to report regarding the effectiveness of quercetin on FBG, FINS, biochemical indicators, oxidative stress markers, inflammatory cytokines, and expression of genes related to the PI3K/PKB insulin signaling pathway in STZ-induced AA broilers.

The remarkable rise of the FBG level observed in STZ-induced broilers was similar as that reported in previous studies with rats [26]. STZ significantly decreased serum FINS levels in rats [27], a finding that was also supported by our data. Hyperglycemic AA broilers had decreased serum FINS levels. Quercetin is a bioflavonoid with the potential to lower plasma glucose, normalize glucose tolerance tests, and reduce oxidative stress markers [28]. On the other hand, quercetin ameliorated the dysfunction of glucose metabolism in STZ-induced diabetic animal models [29].

Flavonoids exhibited different effects on biochemical markers of chickens. AST and ALT contradictory results are observed. “Bashen,” a plant abundant in flavonoids and polyphenols, was added at the rate of 0.5%, 1%, and 2% for 14 weeks into the diets of Taiwan country chickens and did not affect AST and ALT levels [30]. Similar results were reported by An et al. [31], who examined the effects of onion extracts (0.3% and 0.5% levels for 5 weeks) on several parameters of broilers. The results of the present study showed that STZ significantly increased the content of AST (*P* < 0.01) and ALT (*P* < 0.05), and these findings are consistent with that indicated by Ghanbari et al. [32]. AST and ALT are commonly used as clinical marker parameters for the diagnosis of liver diseases; ALT is more specific in liver injury than AST and has been shown to be a good predictor of liver-associated diseases [33]. The current study showed that quercetin dietary supplementation significantly decreased serum AST content of STZ-induced broilers (*P* < 0.01); however, no effect was observed for serum ALT content (*P* > 0.05). It can be therefore concluded that quercetin did not completely ameliorate liver injury in STZ-induced broilers. In contrast, quercetin at the dose of 30 mg/kg body weight significantly lowered serum AST and ALT levels in STZ-induced diabetic rats [32]. Similar results were reported by another researcher, whose supplementation with Cyclocarya paliurus leaves significantly decreased AST and ALT levels in STZ-induced diabetic mice [34]. The variation in results could be partially attributed to the difference in experimental animal models.

NO is a key regulator of vital physiological functions including blood pressure, immune response, and cell signaling. Overproduction of NO is considered a proinflammatory mediator and plays a crucial role in infection and diseases [35]. Furthermore, NO plays the role of a mediator of apoptosis in a variety of cells [36]. One of the evaluation indices for oxidative stress is frequent NO content in biomaterials (serum and tissues). Under conditions of oxidative stress, flavonoids protect NO from superoxide-driven inactivation [37]. The present study showed that STZ and quercetin significantly affected serum and liver NO levels, and these findings were in line with that of Coskun et al. [28], who found that STZ administration significantly increased serum NO concentration. Additionally, quercetin treatment significantly decreased the elevated NO level in STZ-induced diabetic rats [38].

MDA is a highly reactive three-carbon dialdehyde, which is generated as a secondary product of lipid peroxidation. MDA may damage cells at enzyme, protein, and DNA levels, consequently leading to cell death [39]. The degree of oxidative stress is evaluated by measuring levels of MDA in serum and different tissues. In this study, STZ significantly increased serum MDA content and quercetin dietary supplementation at the levels of 0.02% and 0.04% and significantly reduced the elevated MDA. The result was supported by the findings of several other researchers [40, 41]. Oral administration of rutin, a polyphenolic flavonoid at the level of 100 mg/kg of body weight for a period of 45 days, decreased MDA levels in different tissues of STZ-induced diabetic rats [40]. Onion peels and bulbs are a rich source of quercetin. Its supplementation at a rate of 0.5% and 1.0% for 8 weeks lowered MDA levels in the muscles of STZ-induced diabetic rats [41]. Goliomytis et al. [42] reported that quercetin supplementation (0.5 g/kg and 1.0 g/kg diet) linearly decreased the MDA level during storage of meat. Moreover, quercetin administration (15 mg/kg body weight for 8 weeks) significantly decreased the elevated MDA levels in tissues of STZ-induced diabetic rat models [43].

In chicken, antioxidants activated immune function by increasing proliferation of lymphocytes and macrophages [44]. The equilibrium between proinflammatory cytokines and anti-inflammatory cytokines is essential for maintaining health status. Inflammatory cytokines play a key role in inflammation response and antibody secretion in stressed animal models [45]. STZ administration did not significantly decrease content of MCP-1, IL-6, and TNF-*α*; however, quercetin dietary supplementation at the level of 0.04% and 0.06% significantly increased TNF-*α* content compared to that of STZ-induced diabetic broilers. These findings are inconsistent with the results of Jung et al. [41], who reported quercetin reduced TNF-*α* levels in STZ-induced diabetic rats. Green tea polyphenol supplementation (200 mg/kg body weight for 6 weeks) decreased inflammatory cytokine (TNF-*α* and IL-6) levels in rats fed a high-fructose diet [46]. Quercetin supplementation (50 and 100 mg/kg diet) ameliorated plasma IL-6 and TNF-*α* content in STZ-induced diabetic rats [47]. At the same time, the results for IL-6 were different from that of Jung et al. [41], Qin et al. [46], and Gergerlioglu et al. [47]. Mahmoud et al. [48] found that quercetin administration decreased the TNF-*α* level in diabetic rats. Oral administration of quercetin (25 and 50 mg/kg diet) significantly decreased levels of TNF-*α* and IL-1*β* in STZ-induced diabetic rats [49]. Rutin administration further reduced plasma glucose and inflammatory biomarkers (TNF-*α* and IL-6) and restored hepatic antioxidative status in STZ-induced diabetic rats [50]. The findings showed that quercetin may protect immune cells from oxidative damage by inhibiting formation of excessive free radicals and preventing lipid peroxidation.

Oxidative stress is regarded as a major cause of balance disturbance between cellular and metabolic antioxidation and oxidation [51]. This imbalance or loss of cellular redox homeostasis endorsed damage to essential biomolecules such as carbohydrates, proteins, amino acids, lipids, and nucleic acids [52, 53]. These antioxidant defense mechanisms protect cells and tissues against oxidative damage. The major endogenous antioxidant enzyme systems in chicken are SOD, GSH-Px, and CAT [54, 55]. Scarce data exist concerning the effects of quercetin on antioxidant activity in chicken tissues, and there is no study that describes the consequences of quercetin dietary supplementation in STZ-induced diabetic broilers. The present experiment showed that STZ administration had no significant effect on activities of liver SOD, GSH-Px, and CAT, while liver GSH-Px and CAT levels were significantly affected by quercetin dietary supplementation at the level of 0.04%. The results of the present study were different from that of Liu et al., who found that quercetin dietary supplementation at the rate of 0.2, 0.4, and 0.6 g/kg of diet for 8 weeks in laying hens had no effect on liver GSH-Px and CAT activity. On the other hand, quercetin dietary supplementation appeared to increase the SOD level in the liver of laying hens [56]. Furthermore, SOD was increased in serum and breast fillets of broilers when bioflavonoids (5 mg genistein/kg feed, 20 mg hesperidin/kg feed, and 5-20 mg mixture of genistein and hesperidin/kg feed) were fed to chickens for 42 days [57]. In this study, SOD and GSH-Px activities were significantly increased while CAT was significantly decreased in the pancreas of hyperglycemic AA broilers supplemented with quercetin. Our results were different from the findings of Coskun et al. [28] and Adewole et al. [38], in which quercetin supplementation significantly increased activities of SOD, GSH-Px, and CAT in the pancreas of STZ-induced diabetic rats. The variation in results was probably due to the difference in the animal model and dose of quercetin.

The insulin signaling pathway is an important biochemical process which regulates several key biological functions. In insulin-sensitive tissues, flavonoids may regulate glucose uptake and metabolism through cell signaling pathways. The critical pathway linking IRS proteins and metabolic functioning of insulin is that of PI3K/PKB, which is the main process for insulin to play a hypoglycemic role. Flavonoids regulated glucose metabolism in a variety of ways [58], for example, by activating PI3K catalyzed phosphorylation of PIP2, transforming it to PIP3. Then PIP3 binds to PKB/Akt and is activated by other protein kinases. Critical downstream substrates of PKB/Akt are mTOR (involved in the regulation of protein synthesis) [59], GSK3 (involved in the regulation of glycogen synthesis) [60], FoxO1 (involved in the regulation of gluconeogenic and adipogenic genes) [61], and AS160 (involved in glucose transport) [62]. The activated Akt enters the cytoplasm and causes phosphorylation and inactivation of GSK3. The main substrate of GSK3 is glycogen synthase (GS, an enzyme that catalyzes the final step in glycogen synthesis). Glycogen synthesis is inhibited when GSK3 phosphorylates GS. Hence, glucose storage as glycogen is enhanced when Akt inactivates GSK3. In the PI3K/PKB pathway, insulin and insulin receptor substrates activate PI3K through the signal transduction pathway. In the present study, STZ upregulated mRNA expression of IR, PI3K, and PKB/Akt, and certain levels of quercetin dietary supplementation significantly downregulated mRNA expression of IR, PI3K, PKB/Akt, and GSK-3*β* (*P* < 0.01) and also significantly upregulated the expression of IRS-1 mRNA (*P* < 0.05) in STZ-induced broilers (*P* < 0.05). Tzeng et al. [63] found that myricetin (1 mg/kg body weight for 14 days) elevated the expression of IRS and Akt in muscle tissue, whereas it did not significantly affect the expression of IR in insulin-resistant rats. Epicatechin and cocoa polyphenol extract (1-20 *μ*g/mL for 24 hours) enhanced tyrosine phosphorylation and the level of IR, IRS-1, IRS-2, and PI3K, thus activating the PI3K/Akt pathway in HepG2 cells [64]. Green tea extract at 1 and 2 g/kg diet for 6 weeks increased mRNA expression of IRS-2 and GSK-3*β* in the liver and muscle of rats fed high-fructose diets [65]. Moreover, green tea polyphenols increased mRNA expression of IR, IRS-1, and IRS-2 in cardiac muscles of insulin resistant rats [46]. At the same time, grape seed extract increased mRNA expression of IR and IRS-1 in preadipocytes [66]. Relative expression of IRS-1 and IRS-2 were upregulated by total flavonoids of guava and pomegranate leaves in diabetic mice [67]. Sea buckthorn seeds upregulated mRNA expression of IR, PI3K, and PKB protein, indicating an improvement of glucose metabolism in mice fed a high-fat diet [68]. These results indicated that quercetin regulated the PI3K/PKB signaling pathway in AA broilers just like the other flavones, thus improving glucose utilization and glycogen synthesis by promoting PI3K/PKB signaling, thereby lowering serum glucose.

In conclusion, hyperglycemia was successfully induced by STZ in AA broilers. STZ administration decreased the production of insulin and increased blood glucose (hyperglycemia). STZ increased the production of ROS causing oxidative stress, thereby causing abnormalities in glucose metabolism, favoring hyperglycemia. Quercetin dietary supplementation ameliorated the side effects of STZ and improved abnormalities of glucose metabolism induced by oxidative damage, i.e., increased insulin secretion and decreased blood glucose via activating the insulin signaling pathway. It can be concluded that quercetin had a protective effect in hyperglycemic AA broilers induced by STZ through the amelioration of oxidative stress damages.

## Figures and Tables

**Table 1:** The experimental design.

Group	Treatment
A (NC)	Basal diet
B (HC)	Basal diet+STZ
C (quercetin 0.02%)	Basal diet+STZ+0.02% quercetin
D (quercetin 0.04%)	Basal diet+STZ+0.04% quercetin
E (quercetin 0.06%)	Basal diet+STZ+0.06% quercetin

**Table 2:** Composition and nutrient levels of basal diet (air-dry basis).

Ingredients (%)	1-3 weeks	4-6 weeks	Nutrient levels	1-3 weeks	4-6 weeks
Corn	57.50	62.30	ME (MJ/kg)	12.33	12.50
Soybean meal	34.50	30.00	CP (%)	21.75	19.72
Soybean oil	3.00	3.00	Total lysine (%)	1.18	1.04
Fish meal	1.00	1.00	Met+Cys (%)	0.91	0.86
Methionine	0.20	0.20	Ca (%)	1.07	0.96
DCP	1.65	1.70	Total P (%)	0.70	0.68
Limestone	1.52	1.17	Available P (%)	0.46	0.45
Sodium chloride	0.30	0.30			
Multivi. Premix1	0.03	0.03			
Mineral premix1	0.20	0.20			
Choline	0.10	0.10			
Total	100.00	100.00			

Provided per kilogram of diet: vitamin A, 9600 IU; vitamin D_3_, 1200 IU; vitamin E, 24 IU; vitamin K, 0.6 mg; vitamin B_1_, 2.4 mg; vitamin B_2_, 9.6 mg; vitamin B_6_, 4.2 mg; vitamin B_12_, 0.01 mg; biotin, 0.22 mg; folic acid, 0.66 mg; niacin, 30 mg; pantothenic acid, 12 mg; nicotinic acid, 42 mg; Cu (CuSO_4_·5H_2_O), 8 mg; I (KI), 0.7 mg; Fe (FeSO_4_·7H_2_O), 100 mg; Mn (MnSO_4_·H_2_O), 120 mg; Se (NaSeO_3_), 0.15 mg; Zn (ZnO), 100 mg.

**Table 3:** Primers of genes related to the insulin signaling pathway used for mRNA expression level.

Name	Primer (5′-3′)	Sequence no.	Length (bps)
*β*-Actin	Forward: GAGAAATTGTGCGTGACATCA	NM205518.1	152 bp
	Reverse: CCTGAACCTCTCATTGCCA		

IR	Forward: GACTCTCCAACGAACAGGTG	XM001233398.4	156 bp
	Reverse: TCAGCATCTCAATGACCTCAA		

IRS-1	Forward: CAAGTTTGGACAGTGGAGCGT	XM003641084.3	106 bp
	Reverse: CAATGGTTTCCATGGCAATG		

PI3K	Forward: CGGATGTTGCCTTACGGTTGT	NM001004410.1	162 bp
	Reverse: GTTCTTGTCCTTGAGCCACTGAT		

PKB	Forward: CTGATGATGCCAAGGAGATT	NM205055.1	175 bp
	Reverse: TGGTCAGGAGGAGTGATTGT		

GSK-3*β*	Forward: AGCTGTTCCGGAGTTTAGCCTAT	XM004938179.2	151 bp
	Reverse: ACGTTAGGTTCTCCACGAACCA		

**Table 4:** The effects of quercetin dietary supplementation on content of serum inflammatory cytokines in STZ-induced AA broilers (ng L^−1^).

Measurements	NC	HC	0.02% Que	0.04% Que	0.06% Que
MCP-1	286.08 ± 163.01	301.98 ± 57.45	323.12 ± 97.01	383.67 ± 151.97	306.55 ± 57.34
IL-6	113.62 ± 22.51	116.62 ± 10.94	107.42 ± 12.48	107.92 ± 11.45	110.90 ± 10.28
TNF-*α*	209.18 ± 24.14^bc^	180.27 ± 19.41^c^	203.11 ± 26.84^bc^	274.67 ± 38.84^a^	225.19 ± 50.84^b^

Inflammatory cytokines: MCP-1 (monocyte chemoattractant protein-1), IL-6 (interleukin-6), and TNF-*α* (tumor necrosis factor-*α*). NC (normal control broilers), HC (STZ-induced broilers), and quercetin (0.02%, 0.04% and 0.06%) groups. Statistical analysis was performed by one-way ANOVA followed by Tukey's test. Values are expressed as thee means ± S.D. and *n* = 10. Values with different letters are significantly different (*P* < 0.01).

**Table 5:** The effects of quercetin dietary supplementation on activities of antioxidant enzymes in liver of STZ-induced AA broilers (U mg^−1^ of protein).

Measurements	NC	HC	0.02% Que	0.04% Que	0.06% Que
SOD	258.96 ± 22.99	236.76 ± 18.36	256.68 ± 20.36	251.61 ± 24.33	256.15 ± 18.22
GSH-Px	37.51 ± 1.52^a^	37.41 ± 1.41^a^	36.48 ± 1.47^a^	28.85 ± 3.46^b^	36.50 ± 1.69^a^
CAT	18.04 ± 1.57^ab^	19.20 ± 1.88^b^	10.81 ± 1.40^c^	21.02 ± 1.44^a^	18.49 ± 1.10^ab^

Antioxidant enzymes: SOD (superoxide dismutase), CAT (catalase), and GSH-Px (glutathione peroxidase). NC (normal control broilers), HC (STZ-induced broilers), and quercetin (0.02%, 0.04% and 0.06%) groups. Statistical analysis was performed by one-way ANOVA followed by Tukey's test. Values are expressed as means ± S.D. and n = 10. Values with different letters are significantly different *P* < 0.05 (GSH-Px) and *P* < 0.01 (CAT).

**Table 6:** The effects of quercetin dietary supplementation on activities of antioxidant enzymes in pancreas of STZ-induced AA broilers (U mg^−1^ of protein).

Measurements	NC	HC	0.02% Que	0.04% Que	0.06% Que
SOD	79.52 ± 9.98^c^	107.01 ± 21.91^a^	85.49 ± 14.51^b^	95.66 ± 9.50^ab^	77.90 ± 7.68^c^
GSH-Px	4.44 ± 0.43^b^	7.77 ± 1.00^a^	8.30 ± 1.34^a^	6.81 ± 0.70^a^	7.85 ± 0.68^a^
CAT	1.63 ± 0.14^a^	1.19 ± 0.14^ab^	0.68 ± 0.10^c^	0.75 ± 0.17^c^	0.97 ± 0.12^b^

Antioxidant enzymes: SOD (superoxide dismutase), CAT (catalase), and GSH-Px (glutathione peroxidase). NC (normal control broilers), HC (STZ-induced broilers), and quercetin (0.02%, 0.04%, and 0.06%) groups. Statistical analysis was performed by one-way ANOVA followed by Tukey's test. Values are expressed as the means ± S.D. and *n* = 10. Values with different letters are significantly different (*P* < 0.05 (SOD, GSH-Px) and *P* < 0.01 (CAT)).

**Table 7:** The effects of quercetin dietary supplementation on mRNA expression of genes relating to the PI3K/PKB insulin signaling pathway.

Measurements	NC	HC	0.02	0.04	0.06
IR	0.90 ± 0.09^b^	1.22 ± 0.13^a^	0.87 ± 0.23^b^	0.72 ± 0.14^b^	1.02 ± 0.20^ab^
IRS-1	1.16 ± 0.10^ab^	0.85 ± 0.18^b^	1.56 ± 0.63^a^	1.38 ± 0.44^a^	1.09 ± 0.39^ab^
PI3K	0.78 ± 0.18^bc^	1.24 ± 0.14^a^	0.93 ± 0.18^b^	0.59 ± 0.14^c^	0.99 ± 0.39^ab^
PKB/Akt	0.89 ± 0.06^b^	1.27 ± 0.07^a^	0.55 ± 0.21^c^	0.74 ± 0.12^bc^	0.72 ± 0.37^bc^
GSK-3*β*	0.90 ± 0.16^ab^	1.25 ± 0.19^a^	0.77 ± 0.32^b^	0.57 ± 0.14^b^	0.65 ± 0.21^b^

Genes relating to the PI3K/PKB insulin signaling pathway: IR (insulin receptor), IRS-1 (insulin receptor substrate-1), PI3K (phosphatidylinositol-3-kinase), PKB/Akt (protein kinase B), and GSK-3*β* (glycogen synthase kinase-3*β*). NC (normal control broilers), HC (STZ-induced broilers), and quercetin (0.02%, 0.04%, and 0.06%) groups. Statistical analysis was performed by one-way ANOVA followed by Tukey's test. Values are expressed as the means ± S.D. and *n* = 10. Values with different letters are significantly different (*P* < 0.001).

## Data Availability

The data used to support the findings of this study are available from the corresponding author upon request.

## Abbreviations

ALT: Alanine aminotransferase
AST: Aspartate aminotransferase
CAT: Catalase
DM: Diabetes mellitus
FBG: Fasting blood glucose
FINS: Fasting insulin
GSH-Px: Glutathione peroxidase
GSK-3*β*: Glycogen synthase kinase 3
IL-6: Interleukin-6
IR: Insulin substrate
IRS-1: Insulin substrate receptor 1
MCP-1: Chemoattractant protein-1
MDA: Malondialdehyde
NO: Nitric oxide
OS: Oxidative stress
PI3K: Phosphatidylinositol 3-kinase
PKB: Protein kinase B
RNS: Reactive nitrogen species
RO: Reactive oxygen species
SOD: Superoxide dismutase
STZ: Streptozotocin
TNF-*α*: Tumor necrosis factor-*α*.
